# Factors Affecting Postoperative Spinal Epidural Hematoma and the Optimal Order of Vertebral Body Decompression in Multivertebral Microendoscopic Laminectomy

**DOI:** 10.7759/cureus.25404

**Published:** 2022-05-27

**Authors:** Yu Soejima, Takeshi Arizono, Hirofumi Bekki, Akihiko Inokuchi, Teiyu Izumi, Ryuta Imamura, Takahiro Hamada, Kimitaka Nakamura, Mamiko Sakai, Masakazu Yoshimoto, Masatoshi Yamamoto

**Affiliations:** 1 Department of Orthopedic Surgery, Kyushu Central Hospital of the Mutual Aid Association of Public School Teachers, Fukuoka, JPN; 2 Department of Orthopedic Surgery, National Hospital Organization Kyushu Medical Center, Fukuoka, JPN

**Keywords:** postoperative blood pressure, vertebral body decompression, lumbar spinal canal stenosis, microendoscopic laminectomy, postoperative spinal epidural hematoma

## Abstract

Purpose

Symptomatic postoperative spinal epidural hematoma (POSEH) is a complication of spine surgery that occurs infrequently but may cause ongoing serious neurological damage. Due to the narrow entry portal, the risk of hematoma is increased after microendoscopic laminectomy (MEL) compared with conventional open surgery, and the risk might be even higher for multivertebral MEL (m-MEL). The purpose of this study was to clarify the factors affecting the development of POSEH after m-MEL and identify the optimal order for the decompression of vertebral bodies.

Methods

A total of 313 patients who underwent m-MEL from 2016 to 2020 were retrospectively assessed. The cohort comprised 238 patients who underwent two-level MEL, 67 who underwent three-level MEL, and eight who underwent four-level MEL. Symptomatic POSEH was defined as the presence of an epidural hematoma at the surgical site on MRI with symptoms such as lower extremity pain or muscle weakness. We elucidated the incidence of POSEH at each vertebral level and investigated the relationship between POSEH and possible risk factors such as clinical and operative variables.

Results

There were 41 patients in the POSEH group and 272 patients in the non-POSEH group. Seven patients in the POSEH group underwent reoperation. The occurrence of POSEH was related to the number of decompressed vertebral bodies. Patients who underwent L2/3 and L3/4 decompression at the end of the procedure also showed a higher incidence of POSEH at the surgical level.

Conclusion

In patients undergoing m-MEL, treatment of the upper lumbar vertebrae at the end of decompression surgery might be a risk factor for symptomatic POSEH. The incidence of POSEH was particularly increased at L2/3, suggesting that L2/3 decompression should not be performed at last and that careful hemostasis should be applied.

## Introduction

Symptomatic postoperative spinal epidural hematoma (POSEH) is one of the complications of spine surgery. Although the incidence of POSEH is low (0.1%-3%) [1-3], it can cause serious neurological problems such as lower extremity pain, numbness, and paralysis of the lower limbs. A variety of risk factors for POSEH have been identified, including hypertension, multiple intervertebral surgeries, kyphotic alignment, alcohol consumption, and coagulation abnormalities [1,2,4-6].

Microendoscopic laminectomy (MEL) is an established surgical technique, and the number of MEL surgeries is increasing [7]*.* However, due to the narrow portal entry, MEL carries a risk of POSEH [7]; this risk may be even higher in multivertebral MEL (m-MEL). Furthermore, the operative order of vertebral decompression in m-MEL varies depending on the surgeon’s preference, and there is no unified consensus.

The purpose of this study was to clarify the risk of POSEH after m-MEL and investigate the effect of the vertebral body decompression order on the incidence of POSEH.

## Materials and methods

Patients

This retrospective study included 313 patients (191 men and 122 women) who underwent m-MEL for lumbar spinal canal stenosis at our hospital during a five-year period from April 2016 to March 2021; 238 patients had two affected vertebrae, 67 patients had three affected vertebrae, and eight patients had four affected vertebrae. Decompression surgery was performed by one of three spine surgeons when conservative treatment no longer improved each patient’s symptoms. There were no unified regulations regarding the order of decompression of the vertebral levels in m-MEL; the order of decompression in each surgery was performed at the surgeon’s discretion. All clinical evaluations and data were collected from the patients’ charts in accordance with our hospital ethics guidelines (Kyushu Central Hospital, approval number 263). We made skin incisions and put drainage tubes flexibly depending on each decompression case. For example, we made two skin incisions in the case of L4/5 and L5/S1 decompression and put two suction drainage tubes. We put these drainage tubes for the first time after all the decompression has been done. These tubes were placed in all cases and were removed two days postoperatively in the absence of POSEH. Patients underwent MRI if they developed worsened neurological symptoms such as lower extremity pain or decreased lower extremity muscle strength that were not present preoperatively. Symptomatic POSEH was diagnosed when MRI showed a hematoma between two vertebrae that was causing lower extremity symptoms.

Clinical evaluation

We investigated the incidence of POSEH at each intervertebral space after m-MEL (L2/3, L3/4, L4/5, and L5/S1). We evaluated the following data to identify the potential risk factors for POSEH: age, sex, BMI, and American Society of Anesthesiologists Physical Status (ASA-PS). Within the POSEH group, we also examined the differences in perioperative factors between patients who underwent decompression of two vertebrae versus three or more vertebrae. Because the incidence of POSEH was the highest in the L2/3 intervertebral space, followed by the L3/4 intervertebral space, we collected further data on whether the L2/3 intervertebral space was decompressed at the end of the surgery.

Statistical analysis

Data are presented as means and standard deviations. The POSEH and non-POSEH groups were compared using Pearson’s Chi-squared test and Student’s t-test. The JMP software version 14.2.0 (SAS Institute, Cary, NC, USA) was used for statistical analysis. P-values of <0.05 were considered to indicate significant differences.

## Results

**Figure 1:**
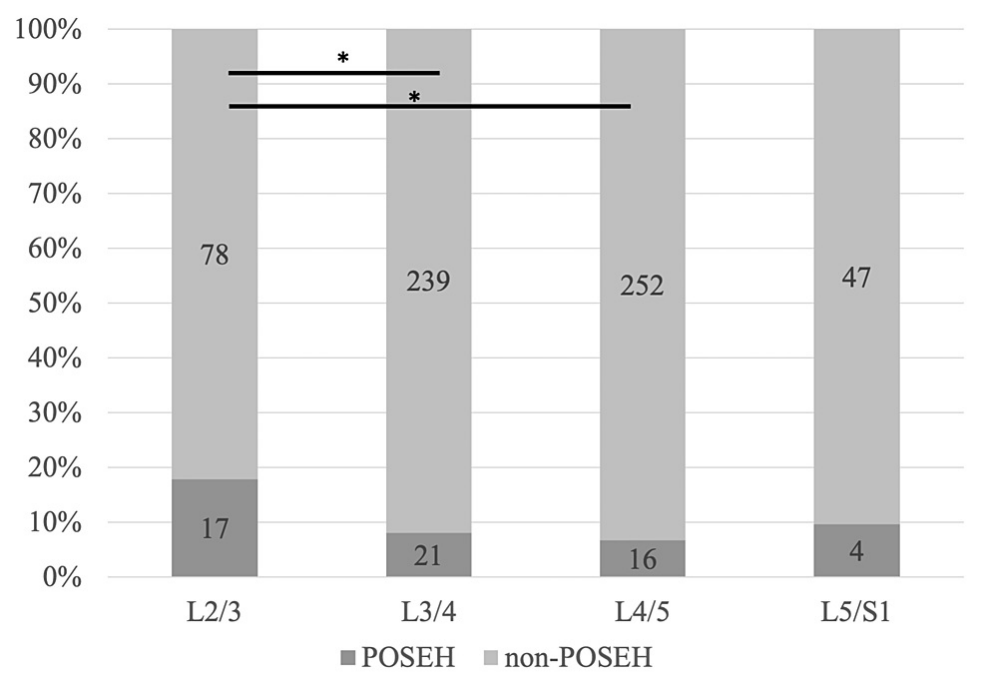
Incidence of symptomatic POSEH in each intervertebral space following multivertebral microendoscopic laminectomy *p<0.05 POSEH: postoperative spinal epidural hematoma

The incidence of symptomatic POSEH at each intervertebral space is shown in Figure 1.

**Figure 2:**
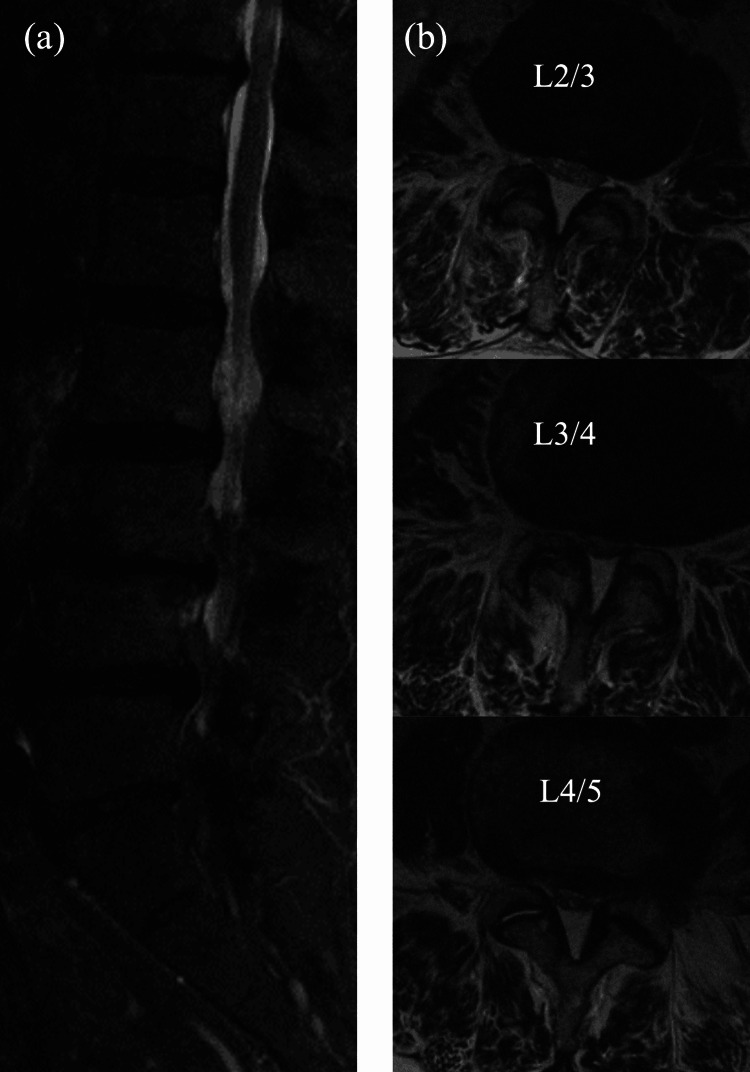
(a) Sagittal plane and (b) axial plane preoperative spinal MRI findings at L2/3, L3/4, and L4/5

**Figure 3:**
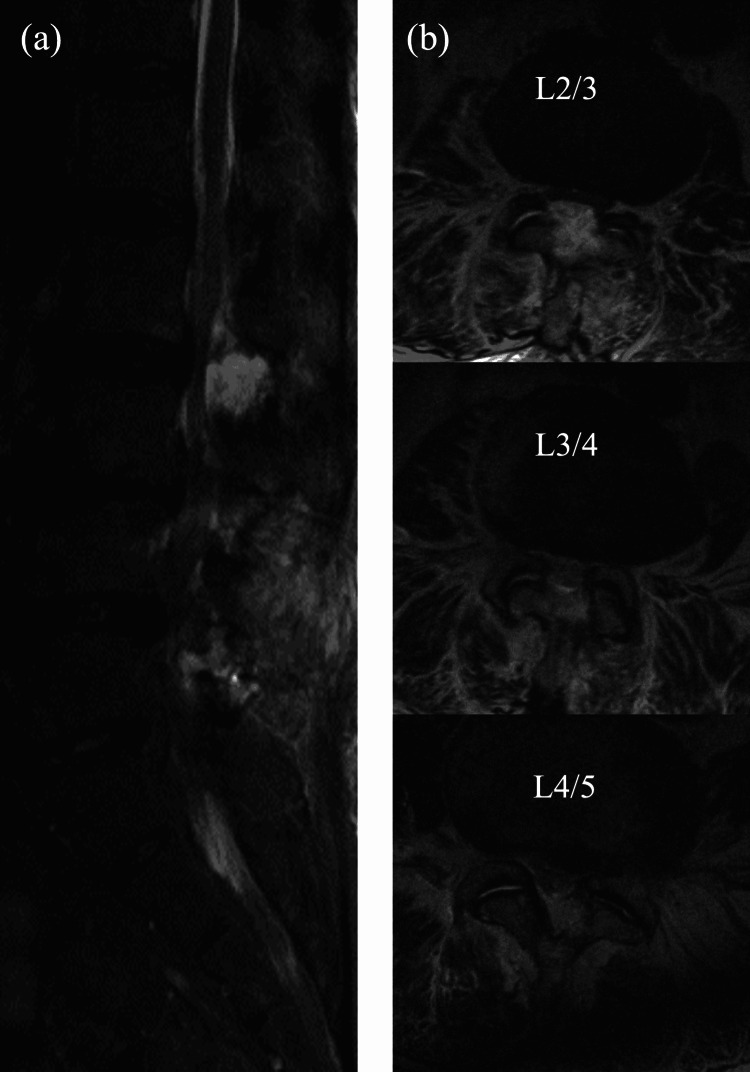
(a) Sagittal plane and (b) axial plane postoperative spinal epidural hematoma at the L2/3 intervertebral space at L2/3, L3/4, and L4/5

Of the 313 patients who underwent m-MEL, 41 patients (13.1%) developed POSEH. The incidence of POSEH was 0% in the L1/2 intervertebral space, 17/95 (17.9%) in the L2/3 intervertebral space, 21/260 (8.1%) in the L3/4 intervertebral space, 16/268 (6%) in the L4/5 intervertebral space, and 4/51 (7.8%) in the L5/S1 intervertebral space. Seven of the 41 patients in the POSEH group underwent revision surgery to remove the hematoma, and the clinical symptoms improved after reoperation in all cases (lower extremity muscle weakness in two cases, bladder-rectal disorder in three cases, and increased lower extremity pain in five cases). The incidence of POSEH was significantly higher in the L2/3 intervertebral space than in the L3/4 and L4/5 intervertebral spaces (Figures 2, 3).

The clinical characteristics are summarized in Table 1.

**Table 1:** Clinical characteristics of patients who underwent multivertebral microendoscopic laminectomy POSEH: postoperative spinal epidural hematoma; BMI: body mass index; ASA-PS: American Society of Anesthesiologists Physical Status; HT: hypertension; n: number of patients; x±y: mean±standard deviation

	POSEH group	Non-POSEH group	P
	(n=41)	(n=272)	
Males/females	11/30	161/111	0.09
Age (years)	72.3±1.7	71.6±0.6	0.72
BMI (kg/m^2^)	24.5±0.6	24.7±0.2	0.68
ASA-PS			
1	4	18	
2	34	217	0.44
3	3	37	
HT	22	51	<0.001
Coagulation problems	23	70	<0.001

The POSEH group comprised 30 men and 11 women with a mean age of 72.3±1.7 years, mean BMI of 24.5±0.6 kg/m^2^, and mean ASA-PS of 1.9. The non-POSEH group comprised 161 men and 111 women with a mean age of 71.6±0.6 years, BMI of 24.7±0.2 kg/m^2^, and mean ASA-PS of 2.07. The clinical characteristics did not significantly differ between the two groups. The operative characteristics are summarized in Table 2.

**Table 2:** Operative characteristics of patients who underwent multivertebral microendoscopic laminectomy *per decompression level n: number of patients; x±y: mean±standard deviation

	POSEH group	Non-POSEH group	P
	(n=41)	(n=272)	
Number of decompressed levels	2.5±0.6	2.2±0.5	0.002
Blood loss volume (g)^*^	17.0±2.61	12.3±1.01	0.10
Operating time (minutes)^*^	75.6±17.5	80.4±20.4	0.15
Blood pressure after extubation (mmHg)	138.3±25.4	139.5±21.4	0.73
Postoperative blood pressure (mmHg)	144.4±23.3	137.3±23.2	0.07

The number of decompressed vertebrae was higher in the POSEH group than in the non-POSEH group (p=0.002). There were no significant differences between the two groups in blood loss volume per vertebral decompression (p=0.1), operative time (75.6±17.5 versus 80.4±20.4 minutes, p=0.15), blood pressure (BP) after extubation (p=0.73), and postoperative BP (p=0.07).

In the POSEH group, there were 23 patients with two vertebrae decompressed and 18 patients with three or four vertebrae decompressed. The operative time per vertebra was significantly increased in patients who underwent decompression of two vertebrae compared with those who underwent decompression of three or four vertebrae (p=0.006) (Table 3). There was no significant difference between groups in terms of blood loss volume or perioperative BP (Table 3).

**Table 3:** Operative parameters of patients who developed postoperative spinal epidural hematoma after MEL at two levels versus three or four levels *per decompression level MEL: microendoscopic laminectomy; n: number of patients; x±y: mean±standard deviation

	Two-level MEL group	Three- or four-level MEL group	P
	(n=23)	(n=18)	
Operating time (minutes)	82.0±14.9	67.3±17.4	0.01
Blood loss volume (g)*	18.4±30.5	15.2±17.2	0.69
Blood pressure after extubation (mmHg)	140.8±24.5	135.1±27.0	0.48

The incidence of POSEH was the highest in the L2/3 intervertebral space, and the details of patients who underwent decompression at L2/3 are summarized in Table 4.

**Table 4:** Operative parameters of patients who underwent microendoscopic laminectomy at the L2/3 level *per decompression level POSEH: postoperative spinal epidural hematoma; n: number of patients; x±y: mean±standard deviation

	POSEH group	Non-POSEH group	P
	(n=17)	(n=78)	
L2/3 treated at the end of surgery	14 (82.3%)	38 (48.7%)	0.01
Operating time (minutes)*	75.4±17.4	79.5±21.7	0.47
Blood pressure after extubation (mmHg)	144.1±30.7	139.6±21.7	0.47
Postoperative blood pressure (mmHg)	155.7±22.7	137.8±24.0	0.006

The L2/3 intervertebral space was decompressed at the end of the surgery in 14 of 17 patients (82.3%) in the POSEH group compared with 38 of 78 patients (48.7%) in the non-POSEH group (p=0.01). There were no significant differences between the two groups in terms of operative time (p=0.47) and BP after extubation (p=0.47). The postoperative BP was significantly higher in the POSEH group than in the non-POSEH group (p=0.006).

The incidence of POSEH among patients who underwent decompression at L2/3, L3/4, or L4/5 at the end of the surgery is compared in Table 5.

**Table 5:** Comparison of patients who underwent decompression at L2/3, L3/4, or L4/5 at the end of the surgery POSEH: postoperative spinal epidural hematoma; n: number of patients; x±y: mean±standard deviation

	POSEH group	Non-POSEH group	Total	P
L2/3	14/17 (82.3%)	38/78 (48.7%)	52/95	0.01
L3/4	13/21 (61.9%)	86/239 (35.9%)	99/260	0.02
L4/5	7/16 (43.7%)	116/252 (46%)	123/268	0.85

Among the patients who underwent decompression of the L3/4 intervertebral space at the end of the surgery, 13 of 21 patients (61.9%) developed POSEH, while 86 of 239 patients (35.9%) did not develop POSEH (p=0.02). Among the patients who underwent decompression of the L4/5 intervertebral space at the end of the surgery, seven of 16 patients (43.7%) developed POSEH, while 116 of 252 patients (46%) did not develop POSEH (p=0.85).

## Discussion

Our study revealed that symptomatic POSEH after m-MEL was most frequently found in the L2/3 intervertebral space and was significantly more common in the upper lumbar vertebrae where decompression was performed at the end of surgery. In spinal endoscopic surgery, the narrow entry portal is thought to affect the incidence of POSEH. Ikuta et al. reported that symptomatic POSEH occurs in about 10% of patients after spinal endoscopic surgery for lumbar spinal canal stenosis [8], which is comparable to the incidence of POSEH in our study.

The morphology of the intervertebral joint and the thickness of the dural sac may play a major role in the high incidence of POSEH at L2/3. The dural sac is the thickest at T9/10 and thinnest at L2/3 [9].^ ^Furthermore, in patients with lumbar spinal canal stenosis, the upper lumbar spine has more advanced sagittalization of the intervertebral joints than usual and a significantly smaller cross-sectional area of the dural sac [10]. The reported risk factors for POSEH include a preoperative MRI cross-sectional area of the dural sac of less than 56 mm^2 ^in the L2/3 intervertebral region [11] and a smaller angle of lumbar lordosis [12]. It has been hypothesized that the intervertebral joints between the upper lumbar vertebrae are more sagittalized than those between the lower lumbar vertebrae, resulting in a larger area of the vertebral arch to be resected in decompression, a wider hemorrhage surface, and an increased risk of bleeding. Although the present study was not able to examine the pre- and postoperative radiographic changes in the vertebral bodies undergoing m-MEL, it is important to understand that the risk of symptomatic POSEH after m-MEL is increased in the upper lumbar spine, especially in the L2/3 intervertebral space.

In the present study, the incidence of POSEH was significantly higher when the last decompression was performed at the L2/3 and L3/4 intervertebral spaces. The current research has reported that preoperative hypertension is involved in the development of POSEH [2] and that post-extubation hypertension and postoperative BP elevations of 50 mmHg or more are likely to cause symptomatic POSEH [3,13]. Among patients with L2/3 intervertebral space decompression, postoperative BP was significantly higher in the POSEH group. These findings indicate that the last vertebra to be decompressed had a shorter period of intraoperative hypotension and shorter time to the attainment of complete hemostasis; therefore, the risk of hematoma was increased due to the increased BP after extubation.

Our study has several limitations. First, we did not verify the preoperative versus postoperative radiographic changes. We did not perform postoperative MRI for non-POSEH groups as control groups. In lumbar spinal canal stenosis, the degree of sagittalization and dural sac stenosis among the superior vertebral bodies can be problematic; however, assessing the changes in vertebral alignment and intervertebral joint decompression in m-MEL may allow for a more quantified hematoma risk assessment. We could not investigate the area of the lumbar spinal canal space, so this is a weak point of this study. We focused on the L2/3 intervertebral space and did not assess the data for L3/4 to L5/S1; the risk factors for symptomatic POSEH may differ in each intervertebral body space. Second, we could not investigate the relationship between the position of the drain and the hematoma. The degree of hematoma drainage may vary depending on the position of the drain [14], and the position of the drain varies between institutions. Third, we were not able to examine the mean intraoperative BP or the time taken for the BP to increase after extubation. More detailed studies of the BP in the postoperative period (after returning to the room and the next day) are warranted.

## Conclusions

The present results suggest that the risk of symptomatic POSEH is increased among the upper vertebrae after m-MEL, and the risk of hematoma may be higher among the last vertebrae to be decompressed especially in the L2/3 and L3/4 intervertebral spaces. Because the incidence of POSEH is particularly high at the L2/3 intervertebral space, care should be taken to achieve adequate hemostasis after the decompression of the L2/3 vertebrae to help prevent symptomatic POSEH.
